# Amniotic Tissues for the Treatment of Chronic Plantar Fasciosis and Achilles Tendinosis

**DOI:** 10.1155/2015/219896

**Published:** 2015-09-27

**Authors:** Bruce Werber

**Affiliations:** ^1^Summit Foot and Ankle Specialists, 9188 E. San Salvador Drive, Scottsdale, AZ 85258, USA; ^2^Amnio Technology, 22510 N 18th Drive, Phoenix, AZ 85027, USA

## Abstract

*Introduction*. Allogeneic amniotic tissue and fluid may be used to treat chronic plantar fasciosis and Achilles tendinosis. This innovative approach involves delivering a unique allograft of live human cells in a nonimmunogenic structural tissue matrix to treat chronic tendon injury. These tissues convey very positive regenerative attributes; procurement is performed with maternal consent during elective caesarian birth. *Materials and Methods*. In the present investigation all patients were unresponsive to multiple standard therapies for a minimum of 6 months and were treated with one implantation of PalinGen SportFLOW around the plantar fascia and/or around the Achilles paratenon. The patients were given a standard protocol for postimplant active rehabilitation. *Results*. The analogue pretreatment pain score (VAS) of 8. By the fourth week after treatment, all patients had significantly reduced self-reported pain. Twelve weeks following the procedure the average pain level had reduced to only 2. No adverse reactions were reported in any of the patients. *Conclusion*. All patients in this study experienced heel or Achilles pain, unresponsive to standard therapy protocols. After treatment all patients noted significant pain reduction, indicating that granulized amniotic membrane and amniotic fluid can be successfully used to treat both chronic plantar fasciosis and Achilles tendinosis.

## 1. Introduction

Heel pain is a common problem that may be due to a variety of soft-tissue abnormalities. Plantar fasciosis is one of the most common causes of heel pain and affects approximately two million people in the US, resulting in one million visits to primary care physicians and foot specialists [1]. Plantar fasciosis is the result of chronic overload from either lifestyle or exercise that promotes tissue degeneration [1]. Similarly, Achilles tendinosis affects both inactive and active individuals and is thought to result from changes in tissue structure [2]. Recent studies have shown that both plantar fasciosis and Achilles tendinitis involve degenerative fibrosis, rather than inflammation. While patients with plantar fasciosis and Achilles tendinitis experience severe, long-term pain, current treatments have limited efficacy, treating only the acute inflammation and pain and failing to address the underlying cause.

In such cases where no effective treatment options exist, engineered* ex vivo* tissues offer promising alternative regenerative therapies. Such tissues can deliver growth factors, fibroblasts, collagen, and extracellular matrix (ECM) on which cells can grow facilitating tissue healing and wound repair. However, many of these tissues are difficult to obtain and can elicit a negative immunogenic response. Embryonic stem cells possess the potential for differentiation into a wide range of cell lineages and hold immense promise for regenerative medicine; however, they are associated with a number of technical difficulties and ethical concerns. Currently, bone marrow (BM) is the most common source of adult stem cells for hematopoietic stem cell transplants and cellular therapies. The mesenchymal stem cells (MSCs) obtained from BM are pluripotent and able to differentiate into many different cell types, including osteoblasts, chondrocytes, adipocytes, neurons, cardiac myocytes, and vascular endothelial cells. BM harvest is an invasive surgical procedure that usually requires general anesthesia or sedation. Additionally, the proliferative potential and differentiation capacity of the BMMSCs from older donors appears reduced. Thus, other sources of stem cells from adult or fetal tissue are sought [3].

The amniotic membrane (AM) or amnion is a tissue of particular interest as a source of readily obtained, multipotent stem cells and factors that promote tissue healing [4]. The AM is the innermost layer of the placenta and consists of a thin epithelial layer, a thick basement membrane, and an avascular stroma. It contains collagen types III, IV, V, and VII and fibronectin and laminin [5, 6]. It also contains fibroblasts and growth factors and has been shown to have unique properties, including the ability to suppress pain, fibrosis, and bacteria and to promote wound healing [7, 8]. The AM contains two cell types of different embryologic origin, specifically amnion epithelial cells, derived from the embryonic ectoderm, and amnion mesenchymal cells, derived from embryonic mesoderm [9]. Recently, the International Society for Cellular Therapy recommended that mesenchymal cells derived from amnion be referred to as amniotic membrane-human mesenchymal stromal cells (AM-hMSCs) [10]. Importantly, amnion is easily obtained after caesarian delivery because the placenta, amniotic fluid, and membrane are typically discarded after childbirth. This procurement avoids the controversies associated with obtaining human embryonic stem cells and BMMSCs. Thus, the use of AM and amniotic fluids (AF) is highly promising innovative allografts and stem cell therapies for degenerative disorders where existing treatments have failed, such as plantar fasciosis and Achilles tendinosis.

AF derived cells are able to replicate rapidly and take 20–24 hours to double in cell number, faster than both umbilical cord stem cells (28–30 hours) and BMMSCs (30+ hours) [11]. The progenitor cells also have a high self-renewal capacity with more than 300 population doublings [12]. In addition, only 30% of MSCs extracted from a child's umbilical cord shortly after birth can be extracted and differentiated. In contrast, the success rate for AF derived MSCs has been close to 100%. Importantly, unlike other multipotent stem cells, particularly those with high self-renewal capacity, the risk of cancer development is low, and AF progenitor cells do not form teratomas* in vivo* [13].

In addition to multipotent stem cells, AF also contains a number of nutrients and growth factors that encourage fetal growth and protection. These factors are highly advantageous in regenerative clinical applications and aid tissue repair. Specifically, AF contains carbohydrates, proteins and peptides, lipids, lactate, pyruvate, electrolytes, enzymes, and hormones, transforming growth factor alpha (TGF-a), transforming growth factor beta 1 (TGF-b1), and fibroblast growth factor (FGF). A recent study demonstrated the effectiveness of FGF in restoring the morphologic and biomechanical properties of injured tendons in rabbits [14].

AF and AM have also been shown to have significant antimicrobial properties, mediated by a-defensins (human neutrophil defensins 1–3), lactoferrin, lysozyme, bactericidal/permeability-increasing protein, calprotectin, secretory leukocyte protease inhibitor, psoriasin, and cathelicidin [15]. Human beta-defensin-2 is another natural antimicrobial peptide present in the AF that may account for much of its antimicrobial activity [16]. Furthermore, lactoferrin, a glycoprotein secreted into the AF by neutrophils and amniotic cells, has bacteriostatic and bactericidal activity [17]. Human AF also contains factors known to minimize scarring. Hyaluronic acid (HA) is abundant in AF and fetal HA is thought to inhibit collagen deposition to prevent fibrotic tissue formation [18, 19]. In recent studies addressing the effect of AF on proteases important for wound healing, human AF was shown to enhance collagenase activity but to inhibit activation of hyaluronidase, elastase, and cathepsin [20, 21].

Due to its regenerative, anti-microbial and anti-scarring properties the amnion has been used as an effective wound dressing and as a graft for skin wound coverage. Several studies have highlighted the low immunogenicity of human amniotic epithelial cells following transplantation into human volunteers. For example, no signs of acute rejection were observed after amnion was transplanted into subcutaneous pouches in normal human volunteers [22]. Following transplantation of amniotic tissues HLA antibodies are absent in serum samples [23]. In addition, amnion surface epithelial cells do not express HLA-A, HLA-B, HLA-C, or HLA-DR or b2-microglobulin [23, 24]. This, at least in part, explains why amniotic tissues can be used successfully as a skin graft without concern for tissue typing and matching of the donor to the host [8]. This lack of immunogenicity has been described in numerous clinical studies and is termed immune privilege [25]. Collectively, these studies suggest that acute immune rejection does not occur after transplantation of human amniotic epithelial cells, and granulized AM and AF (gAM-AF) are a suitable treatment option for all patients, even those who are severely immunocompromised.

## 2. Allograft Procurement

In the present study, patients experiencing heel pain caused by chronic plantar fasciosis and Achilles tendinosis and who were unresponsive to standard therapies for a minimum of 6 months were treated with PalinGen SportFLOW (Amnio Technology, llc. Phoenix, AZ) to promote tissue repair and regeneration. The PalinGen SportFLOW allograft was generated from human amniotic membrane and amniotic fluid (hAM-AF), harvested from females undergoing elective caesarian section. PalinGen SportFlow is a human allograft and is processed and packaged at an FDA registered tissue bank accredited by the American Association of Tissue Banks (AATB). PalinGen SportFLOW is regulated by the FDA under Title 21 Part 1271 Section 361 of the Public Health Service Act. Tissues were tested extensively to ensure the absence of communicable diseases and other abnormalities. After testing, the tissues were aseptically processed and cryopreserved to preserve cell viability. Cryopreservation of the hAM-AF yielded a multifactorial tissue matrix containing viable pluripotent mesenchymal stem cells, fibroblasts, keratinocytes, epithelial cells, cytokines, proteins, growth factors, and multipotent cells, all required for fetal growth and development and able to stimulate tissue repair and regeneration. In this study, allografts were used to create a microenvironment suitable for regeneration of tendons and fascia that had become chronically thickened due to abnormal function and healing. Allograft was also used as a potent anti-inflammatory and to create the appropriate conditions in which to drive poorly formed tendons and fascia to a normal state.

The PalinGen SportsFLOW allograft most important components are a wide spectrum of growth factor proteins, that are, VEGF, TGF-beta1, EGF, PDGF-AA, PDGF-BB, FGFb, extracellular matrix (cryofractured amnion membrane) and amniotic fluid derived cells.

Amnion donors were subject to a thorough prescreening process performed by the Medical Director. Eligibility was confirmed through behavioral risk assessment, medical history, hematology, and communicable disease testing. Procurement of the amnion tissues was done with an aseptic recovery technique during cesarean section, using standard sterile techniques. Procurement of hAM-AF does not require fetal death, and its recovery was performed with maternal consent during an elective caesarian section live birth.

## 3. Patients, Methods, and Techniques

Chronic plantar fasciosis patients were chosen from a pool of patients with chronic heel pain that had failed a variety of noninvasive therapies, including custom and/or prefab orthotics, stretching, steroid injections, physical therapy, and night splints (used for 1-2 hours in evening with leg extended). These patients additionally had a thickened fascia on diagnostic ultrasound of at least 4.0 mm.

## 4. Plantar Fascia Technique

1. ZimmerWave radial pulse therapy was applied to the painful area, typically 1500 pulses, at 10 hertz and 110 mJ.
2. The plantar fascia was visualized under ultrasound imaging, and the area was aseptically prepared with Betadine or alcohol.
3. 0.5 mL of PalinGen SportFLOW and 0.5 mL of 1% lidocaine were drawn into a 3 mL syringe with a 22-Gauge needle.
4. Observing the plantar fascia under ultrasound guidance, the approach was from medial to lateral. The needle was directed to the superior surface of the plantar fascia, not directly into the plantar fascia, and 0.3 mL was deposited along the medial and central bands of the fascia.
5. The needle was then redirected to the plantar aspect of the plantar fascia and another 0.3 mL was deposited along the fascial band.
6. The needle was then redirected towards the central plantar calcaneal bursa between the medial and lateral tubercles and the remainder of the allograft was implanted.
7. The patient was instructed to stretch every 30 minutes with a traditional runners calf stretch during waking hours.
8. Patients were also instructed to wear lace up stable shoes, to minimize time being barefoot and to minimize wearing flip-flops or slip-on shoes.
9. No anti-inflammatory medication was taken for 3–6 weeks.
10. No ice was applied to the affected area.

Achilles tendinopathy patients were chosen from a pool of patients with Achilles pain, who were unresponsive to a variety of therapies including stretching, physical therapy, and modified shoe gear and in some cases low energy radial pulse therapy. Imaging confirmed that there was no rupture using either MRI or diagnostic ultrasound.

## 5. Achilles Tendon Technique

1. ZimmerWave radial pulse therapy was applied to the painful area, typically 1500 pulses, at 10 hertz and 110 mJ.
2. The Achilles tendon was visualized under ultrasound imaging, and the area was aseptically prepared with Betadine or alcohol.
3. 1.0 mL of PalinGen SportFLOW and 1.0 mL of 1% lidocaine were drawn into a 3 mL syringe with a 22-Gauge needle.
4. Observing the Achilles tendon under ultrasound guidance, the needle was directed along the paratenon starting on the medial aspect, not into the tendon substance, and 0.5 mL was deposited along the medial aspect of the tendon. The needle was then redirected to the posterior aspect of the tendon and the remainder was deposited along the lateral aspect.
5. Patient was instructed to stretch every 30 minutes with a traditional runners calf stretch during waking hours.
6. Patients were also instructed to wear lace up stable shoes, to minimize time being barefoot and to minimize wearing flip-flops or slip-on shoes.
7. No anti-inflammatory medication was taken for 3–6 weeks.
8. No ice was applied to the affected area.

## 6. Results

In total, 44 patients experiencing chronic plantar fasciosis and Achilles tendinosis, with a mean age of 55.1 and 47.7 years, respectively, who were all unresponsive to multiple standard therapies for a minimum of 6 months, were treated with one implantation of PalinGen SportFLOW around the plantar fascia and/or into and around the Achilles paratenon. Following treatment they were instructed to wear laced shoes and perform posterior muscle group stretching exercises or rolling pin massage instructions. No changes were made to their exercise routines.

The visual analog scale (VAS) is an instrument used to quantify the level of pain reported by patients. The VAS ranges from 0 to 10 with 0 representative of no pain, 1–3 indicating mild pain, 4–6 indicating moderate pain, and 7–10 representative of severe pain. Preoperative pain was self-reported as severe in all patients, with a mean of 8.2 (Table 1). Changes in self-reported pain were monitored every 2 weeks for 12 weeks after procedure. Changes in pain over time were statistically determined using the Freidman nonparametric repeated measures ANOVA with Dunn's post hoc test for multiple comparisons.

**Figure 1:**
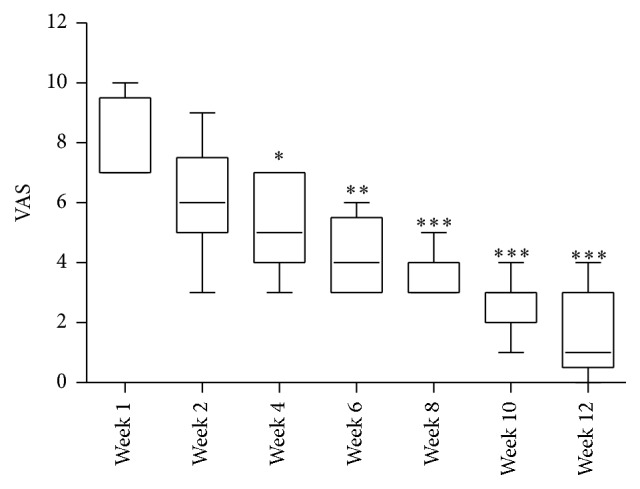
Time course self-reported postoperative visual analogue pain score (VAS) from patients with plantar fasciosis, showing median and minimum and maximum scores, where ^*∗∗∗*^ *p* < 0.001 versus preop (week 1) VAS.

For patients experiencing plantar fasciosis there was a significant improvement in pain scores in all patients by postoperative week four (*p* < 0.05, Figure 1), with a mean pain score of 5.2 (Table 1) indicative of moderate pain. By postoperative week 10 the pain scores were markedly reduced (*p* < 0.0001, Figure 1) and the average self-reported scores indicated that the majority of patients experienced only mild pain.

**Figure 2:**
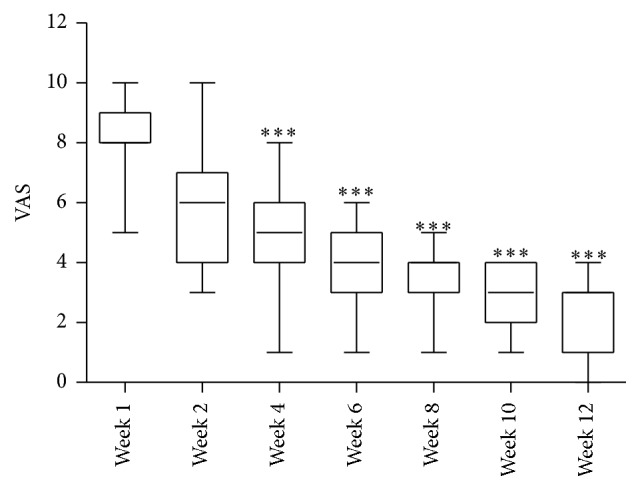
Time course self-reported postoperative visual analogue pain score (VAS) from patients with Achilles tendinosis, showing median and minimum and maximum scores, where ^*∗∗∗*^ *p* < 0.001 versus preop (week 1) VAS.

Similar results were observed in patients experiencing Achilles tendinosis, and all patients gave self-reported pain scores not higher than moderate pain by postoperative week 6, with an average pain score of 4.7 (Table 1) ranging from 1 to 6 (Figure 2). By 12 weeks after treatment the average pain score had reduced to only 2.3 (Table 1) indicating that the majority of patients were experiencing mild pain. Therefore, after treatment with granulized amniotic membrane and amniotic fluid pain was significantly reduced compared to preoperative pain, with the majority of patients reporting only mild pain.

## 7. Discussion and Conclusion

Heel pain is a common problem that may be present in 15% of patients presenting to their primary care physician [26]. In this study we show for the first time to our knowledge that a single injection of hAM-AF allograft is sufficient to significantly reduce heel pain caused by plantar fasciosis and Achilles tendinosis. At the end of the study all patients showed a significant improvement in pain, and on average self-reported pain had reduced from severe to mild. Our findings suggest that amniotic allografts create the appropriate environment needed to promote tissue repair and healing in complex soft-tissue disorders such as plantar fasciosis and Achilles tendinosis.

Plantar fasciosis is the most common cause of inferior heel pain and is often due to repetitive mechanical stress, producing microtears and inflammation of the fascia and perifascial soft tissues. The condition is commonly seen in individuals who are susceptible to injury such as runners and obese patients [27]. To date there is no definitive treatment proven to be the best option for plantar fasciosis. Treatment is patient dependent and commonly requires a combination of different therapies to successfully alleviate symptoms [28]. In many cases patients do not respond to current treatments and symptoms persist. This is likely due to the fact that plantar fasciosis is not simply the product of mechanical stress and is actually the result of a number of contributing factors associated with aberrant tissue development and healing. Factors include enthesopathy in association with seronegative spondyloarthropathies, such as ankylosing spondylitis, Reiter syndrome, or psoriatic arthritis [29]. Findings from MRI studies have shown a number of other tissue abnormalities associated with plantar fasciosis, including plantar fascial thickening and intrafascial edema [29].

Achilles tendinosis is also a common cause of heel pain in a sport-active population and is responsible for reduced physical performance and increased severe pain over several years [30]. Despite being associated with mechanical stress, recent studies have shown that this pathology also affects an older population with less involvement in sporting activities, suggesting that tissue degeneration, in some cases age-associated, contributes to its pathogenesis. Recent reports also highlight the heterogeneity of Achilles tendinopathy pathogenesis and have identified multiple synergistic risk factors including genes, age, circulating and local cytokine production, sex, biomechanics, and body composition [31].

Current conventional treatments for heel pain include physical therapy, rest, stretch exercise, nonsteroidal anti-inflammatory drugs (NSAIDs), and steroid injections. Steroid injection is one of the most popular options [32]; however, it may produce serious side effects such as a recognized risk of subsequent plantar fascia rupture [33]. Consequently, treatments that only address the symptoms of plantar fasciosis and Achilles tendinosis are often unsuccessful, and treatments able to stimulate wound healing are highly sought.

Provision of factors that provide a regenerative stimulus is an emerging treatment strategy which aims at alleviating chronic tendinopathies characterized by a poor healing ability. Recent studies have shown that provision of platelet rich plasma (PRP)—rich in platelet derived growth factors—can provide a local regenerative stimulus for tissue healing. Achilles tendinopathy patients receiving PRP injections showed significant improvements after treatment; however, these improvements took several months to occur [34]. MSCs are an emerging alternative option to promote tissue regeneration. Recently, several studies in animal models have shown that administration of hMSCs can improve healing in tendon injuries. Specifically, hMSCs can support tendon healing through better vascularization, larger deposits, and better organization of the extracellular matrix [35]. Although overall this treatment procedure may be clinically safe, cartilage and bone formation at the implantation site is an expected adverse event [35]. In addition procurement of hMSCs is associated with invasive surgical procedures and ethical concerns.

Amniotic tissue allografts are also associated with soft-tissue repair and regeneration. Specifically, recent studies have shown that amniotic allografts contain angiogenic growth factors that promote amplification of angiogenic cues by inducing endothelial cell proliferation and migration to promote the formation of blood vessels* in vivo* [36]. Such grafts offer promising stem cell therapies with the potential to promote revascularization and tissue healing within poorly vascularized, nonhealing wounds. In addition, amniotic allografts are not associated with problematic procumbent procedures and contain additional factors with anti-inflammatory and anti-microbal properties. However, preservation of these properties during processing remains a challenge.

To date, the efficacy of amniotic tissue allografts in rescuing chronic heel pain has not been demonstrated. In the present study, cryopreserved (PalinGen SportFLOW) hAM-AF was injected into the tissues of patients who experienced severe heel pain and who were unresponsive to existing therapies. Significant improvements in pain were observed 4 weeks after treatment in all patients, with almost complete pain recovery in many patients by the end of the study. Our observations suggest that cryopreserved hAM-AF mediates the biological properties required for effective and rapid tissue healing and repair. Our findings support the use of PalinGen SportFLOW allograft as a promising therapy for plantar fasciosis and Achilles tendinosis and other soft-tissue disorders associated with deficiencies in the normal wound healing processes.

## Figures and Tables

**Table 1:** Baseline statistics and time course postoperative visual analogue pain scores (VAS) for patients with either plantar fasciosis (PF) or Achilles tendinosis (AF). Data is presented as mean with SD. ^*∗∗∗*^ *p* < 0.001 versus preop VAS (mean age compared with Mann-Whitney *U*-test).

	Patient age	Preop	Week 2	Week 4	Week 6	Week 8	Week 10	Week 12
PF	55.11 ± 5.9	8.1 ± 1.4	6.2 ± 1.8	5.2 ± 1.6	4.2 ± 1.2	3.6 ± 0.7^*∗∗∗*^	2.5 ± 0.9^*∗∗∗*^	1.5 ± 1.4^*∗∗∗*^
AT	47.69 ± 3.3	8.2 ± 1.2	6 ± 1.9	4.7 ± 1.6^*∗∗∗*^	4 ± 1.0^*∗∗∗*^	3.6 ± 0.9^*∗∗∗*^	2.9 ± 1.0^*∗∗∗*^	2.3 ± 1.3^*∗∗∗*^
